# Neurologic dance training (NDT) as supportive care for the physical symptoms of cancer: a systematic review and meta-analysis

**DOI:** 10.1080/28352610.2025.2594717

**Published:** 2025-12-04

**Authors:** Lise Worthen-Chaudhari, Maya Patel, Kristen Lantis, Shreya Ghanekar, Sarah Addison, Esther Jung, Whitney Luke, Sara Faithfull, Jewel Crasta, Maryam B. Lustberg

**Affiliations:** a College of Medicine, Department of Physical Medicine and Rehabilitation, The Ohio State University, Columbus, OH, USA; b Department of Medicine, Division of Internal Medicine, Washington University, St Louis, MO, USA; c Discipline of Radiation Therapy, School of Medicine, Trinity College Dublin, the University of Dublin, Trinity Centre for Health Sciences St. James’s Hospital Campus Dublin 8, Dublin, Ireland; d Transforming cancer OUtcomes through Research (TOUR), School of Cancer & Pharmaceutical Sciences, King’s College London, London, UK; e College of Medicine, School of Health and Rehabilitation Sciences, The Ohio State University, Columbus, OH, USA; f Yale Cancer Center, Yale University, New Haven, CT, USA

**Keywords:** Cancer survivorship, dance, music, rehabilitation, physical medicine

## Abstract

**Objective:**

This systematic review and meta-analysis assessed evidence regarding dance as nonpharmacologic treatment for the physical symptoms of cancer survivorship.

**Data sources:**

PubMed, Embase, Scopus.

**Study selection:**

Following PRISMA guidelines, two reviewers independently screened and assessed data. Randomized controlled trials (RCTs) measuring effects of dance on physical symptoms among survivors were included.

**Data extraction:**

We extracted characteristics of included studies (e.g., cancer type, dance technique) and interventions (e.g., frequency, intensity, time, type).

**Data synthesis:**

Twelve RCTs met criteria for inclusion. Dance genres studied included Argentine tango, Greek traditional dance, dance movement therapy, belly dance, ballroom, Latin dance, quang chang wu, The Lebed Method™, and improvisation. Risk of bias was low overall. Meta-analyses revealed a favorable effect on patient-reported physical health with a standardized mean difference (SMD) = 1.51, 95% confidence interval (CI) = [0.23, 2.79], *p* = 0.03; gastrointestinal dysfunction SMD = 0.53, CI = [−0.27, 1.34], *p* = 0.08; fatigue SMD = 0.35, CI = [−0.18, 0.88], *p* = 0.12; stress SMD = 1.43, CI = [−1.48, 4.34], *p* = 0.17; walking endurance SMD = 0.37, CI = [−1.43, 2.17], *p* = 0.23; physical activity level SMD = 0.82, CI = [−0.99,2.63], *p* = 0.24; and sleep SMD = 0.44, CI = [−0.89, 1.77], *p* = 0.29.

**Conclusion:**

Dance interventions show potential to alleviate physical symptoms due to cancer or cancer treatment. More work is needed to standardize delivery of dance as a physical rehabilitation option within survivorship care.

Cancer detection and treatment continue to improve such that the number of individuals surviving with cancer currently exceeds 32 million individuals worldwide [1,2]. Despite these care advances, cancer and its treatment continue to cause debilitating physical sequelae [1,3] with limited treatment options [4,5]. Regular exercise remains the leading recommended treatment for these physical symptoms [3] despite evidence that individuals with cancer are less motivated to engage in exercise than in other types of physical activity [6]. Physical activity that takes the form of dance has the potential to distinctly motivate therapeutic physical activity practice for individuals with cancer and other chronic physical disabilities [6–8], and improve function through neurophenomena including but not limited to auditory–motor entrainment [9–11]. Although the psychotherapeutic use of dance movement therapy (DMT) is a known treatment for the psychosocial symptoms of cancer [12–14], therapeutic treatment of the physical effects of cancer through dance–based intervention remains underexplored.

The physical sequelae of cancer present as disabling and unwanted dysfunction within the brain and body throughout the stages of cancer (Figure 1). Clinical identification of physical symptoms relies heavily on patient–reported outcome measures (PROs) of pain, deconditioning, fatigue, neuropathy, nausea, and weakness. These PROs serve as surrogate measures of underlying conditions created by the progression and treatment of cancer such as sensorimotor and cognitive dysfunction, dual–task deficits, cardiotoxicity, pulmonary toxicity, infertility, lymphedema, aerobic deconditioning, fatigue, nausea, gastroenterological distress, fibromyalgia, osteoporosis, and metabolic changes causing involuntary weight loss or gain [3,5,15–18]. Mechanistically, physical morbidity associated with cancer progression and treatment can be linked to compromised neuromuscular biomechanics [8,19–23], sensorimotor control [24–26], mechanoreceptor encoding [25,27], biomarkers of inflammation and neurotoxicity [28,29], and brain structure and activity [17,30–32]. In some cases, the cancer progression causes these dysfunctions, however cancer treatments such as chemotherapy, radiation, and other life–saving measures also contribute heavily to physical morbidity [5]. Finally, it is important to note that, while cancer–related physical sequelae are measured as distinct from the psychosocial effects of cancer, there is an interaction between the domains [16] such that successful intervention in physical health stands to improve psychosocial outcomes and vice versa.

Dance is a subset of motion behavior [33] that invokes physiologic neurophenomena known or hypothesized to facilitate recovery from neural damage [8], such as entrainment, chronoception, spatial cognition [34], biomarker regulation [35,36], and neural synchrony [37]. Importantly, these same mechanisms may be disrupted in chronic neurologic and oncologic conditions, wherein individuals often experience persistent neurocognitive and sensorimotor sequelae (e.g., chemotherapy–induced neuropathy, cancer–related fatigue, cognitive impairment). Evidence from recent trials among breast cancer survivors demonstrates that dance training (e.g., Adapted Tango) improves dual–task function and reduces cognitive load, even several weeks post–intervention, supporting the potential for durable neural recovery [38]. Our overarching hypothesis is that dance behavior organizes the human dynamic system to self–organize [39–41], systemically, in ways that potentially improve chronic neurologic and oncologic conditions. Within the current systematic review and meta–analysis, we evaluate existing evidence related to this hypothesis.

When dance interventions are structured using evidence–based models for medical activity design, such as the *frequency, intensity, time,* and *type* (FITT) principle [42], then they represent neurologic dance training (NDT) [8] candidates with potential to improve physical and/or cognitive outcomes, in addition to psychosocial health, across the continuum of care [7,8,43,44]. We define NDT as movement training techniques adapted for persons with mobility deficits that deliver physical activity in the context of multisensory integration [9,10,45,46] and creative engagement [7]. Essential components of NDT include multisensory integration (e.g., visual, auditory, vestibular, proprioceptive), patterned movement sequences, movement exploration (i.e., improvisation), engagement of the imagination during motor performance (e.g., via analog learning). Dance behavior is often accompanied by music in a way that activates the brain’s ability to predict and adapt to auditory information, however, musical accompaniment is not required as it is possible to practice the art of dance in silence (e.g., contact improvisation, Manipuri Indian classical dance, Appalachian flatfoot dance). Unlike recreational dance or structured movement therapies, NDT explicitly integrates sensorimotor learning strategies such as trial–and–error [40] exploration via movement improvisation (versus rote repetition), autonomy support [47–49], analogy learning [50], and auditory–motor cueing [11,51], thereby distinguishing it from other physical activity–based supportive care interventions. A growing evidence–base supports the efficacy of dance–based intervention to slow neurodegenerative change among individuals with Parkinson Disease [52–55]. The potential to improve physical morbidity resulting from cancer and its treatment remains underdeveloped.

Prior reviews of dance for survivorship hint at a positive effect on physical symptoms, however, a comprehensive review of evidence remains to be conducted. Most prior systematic reviews and meta–analyzes report positive physical effects of dance–based intervention that is limited in scope in some way that challenges application. For instance, reviews, by Fatkulina et al. (2021), Brandt et al. (2015), and Archer et al. (2015) [12,14,56] reported effects of DMT psychotherapeutic interventions on limited patient–reported outcomes (PROs) of physical health among survivors, such as physical activity level and fatigue [12,14,56] but did not review dance–based intervention outside of DMT. In another example, Nelson et al. (2023) limits the type of intervention reviewed to community–based dance, not including DMT, in their report of a positive effect on functional capacity and fatigue among survivors [57]. In a third example, Rudolph et al. (2018) [58] reviewed the effects of ballroom dance, specifically, on physical fitness and fatigue among survivors, finding that only 1 RCT met criteria at that time. Other reviews limit the scope of outcomes reviewed outside of the psychological realm. For example, Boing et al. (2017) review dance–based interventions, including but not limited to DMT, but limit analysis of effect on physical symptoms primarily to upper extremity outcomes such as strength and range of motion (Boing et al. 2017) [59]. Abu–Odah et al. (2024) apply an interesting approach by separating dance movement interventions (DMIs) from DMT interventions and reporting effects of those interventional subgroups on a host of physical symptoms–such as pain severity, pain interference, fatigue, sleep, and hand grip strength. Abu–Obah et al. (2024) provide a strong review with a valid approach, however, the omission of physical outcomes related to chemotherapy exposure, such as dual–task function and neuropathy, limit application of results in survivorship care [60]. While these excellent prior reviews inform the current one, we note the gap in literature that remains. To fill this gap, we aim to address the effect of dance–based intervention, including any type of intervention involving movement–to–music (e.g., community–based techniques, DMT practice), on the full scope of physical sequelae associated with survivorship. Toward this end we clarify the scope of physical symptoms per phase of survivorship in Figure 1.

This systematic review and meta–analysis assess the evidence regarding the effects of dance–based interventions on physical symptoms among survivors of any cancer type, stage, or phase. We include techniques from medically prescribed dance (DMT) to community–based dance to consider how NDT might fit as a community–based pathway for physical rehabilitation and wellness across the continuum of survivorship care. As stated, dance does not require musical accompaniment, however, to focus on the most concrete examples of dance trialed as physical medicine, we limit our consideration to dance forms that involve moving to music [61,62], also referred to in the scientific literature as rhythmic rehabilitation [63]. Finally, we include both choreographed and improvisational dance practices. This review and meta–analysis aimed to answer the question of whether evidence supports consideration of NDT [8] as a candidate physical medicine modality for survivors of cancer [8,38,64].

## Methods

We conducted a systematic review and meta–analysis following Cochrane recommendations and Preferred Reporting Items for Systematic Reviews and Meta–Analyzes (PRISMA) guidelines. We did not register nor publish the protocol prior to conducting it.

### Information sources:

We performed literature searches in PubMed, Embase and Scopus in January of 2025, with no limit on date of publication.

### Search strategy:

We used the following search terms: cancer* or oncolog* or tumor* or tumor* or neoplasm* AND danc* or dance therapy or music or music therapy in all three database searches. In PubMed, we also used the following MeSH terms: dancing, dance therapy, music, music therapy, and neoplasms. In Embase, we used the following Emtree terms: dancing, dance therapy, music, music therapy, and malignant neoplasms. We detail our search strategies per database in Supplementary Table A.

### Eligibility criteria:

We included articles if they were randomized controlled trials (RCT) that measured the effect of dance intervention on cancer–related physical sequela. Only studies that included adult participants (≥18 years) were eligible. We included RCTs that reported physical outcomes as primary, secondary, or exploratory endpoints, provided that sufficient data were reported to allow analysis. We excluded articles if interventions studied did not meet our definition of dance (e.g., listening to music without moving; moving without attention to an auditory rhythm); were reported in abstract form only; or were unavailable in English including via Google Translate or other free services. We used review articles to identify studies missing from our initial search but did not include these review articles in data extraction. We excluded feasibility or pilot studies to prioritize methodologically rigorous trials and avoid duplication, although we acknowledge that such studies may provide preliminary insights into intervention implementation and tolerability. We took the following steps to ensure appropriate effect estimates by avoiding double–counting of data. We omitted publications that published preliminary results from a study in favor of final publication of results and we used National Clinical Trial (NCT) numbers to help identify these instances. When RCTs identified for inclusion featured more than one arm we performed pairwise analysis with the single most relevant comparison among the possible arms. When available, we selected routine treatment as the comparison arm; when no standard of care arm was evaluated, we selected the comparison therapeutic activity that was least like dance. For cross–over trials, we included only data from the main comparison and did not include data from cross–over periods.

We define cancer–related physical sequelae as the adverse physical effects that arise directly from cancer, its treatment, or the intersection of these factors with a person’s overall health such as but not limited to endurance, pain, stress, gastrointestinal dysfunction, sleep, and/or fatigue. We included studies that measured these adverse physical effects with patient–reported outcomes (PRO) (e.g., neuropathy scales, fatigue inventories); clinical tests of function (e.g., 6 minute walk test of endurance, timed–up–and–go test of mobility); and/or quantified biophysical outcomes (e.g., postural sway, circulating cortisol concentration).

### Study selection:

We managed screening using Covidence systematic review software (Veritas Health Innovation, Melbourne, Australia; accessed [20 January 2025]. Two investigators reviewed titles and abstracts retrieved by the search once Covidence removed duplicates. When agreement on inclusion was unanimous, we included publications for full text review. When there was disagreement, the lead investigator (first author LWC) reviewed the title and abstract in question and made the final decision regarding inclusion or omission. Two investigators completed full text reviews of included publications.

### Data extraction:

After applying the exclusion criteria, we performed data extraction on the remaining articles. We used the PICO framework (i.e., participant, intervention, comparison, and outcome) [65] to guide abstraction of data elements. Participant characteristics extracted included cancer type and stage inclusion criteria as well as and mean(SD) age of the enrolled cohort. To define intervention characteristics, we extracted data about the frequency, intensity, time, and type of interventional activity (FITT) [42] and longitudinal period of activity participation to characterize the dose of dance behavior investigated per study. Additionally, we characterized the environment in which the study was performed including the country in which the study was performed, qualifications of the instructor, instructional environment in terms of group characteristics and setting (e.g., hospital, community), and whether the dance form had been adapted systematically from another dance form (e.g., Adapted Tango). We summarized comparator interventions per RCT in terms of usual care or active control; in the case of an active control intervention comparator, we extracted the type of activity that dance was compared to (e.g., exercise, music listening). We performed content analysis to identify physical health outcomes measured across studies. We summarized resulting variables per study with statistical significance level and effect sizes (i.e., Cohen’s D) calculated where possible. Finally, we assessed whether included studies reported adverse events in their results.

### Risk of bias assessment:

We used the Cochrane Risk–of–Bias Tool for Randomized Trials (RoB−2), to assess all of the included RCTs [66]. Two authors (SG and LWC) independently evaluated the 5 domains of bias: randomization process, deviations from intended interventions, missing outcome data, outcome measurement, and selection of reported results. We judged each domain as presenting with either a low, high, or unclear risk of bias.

### Quality assessment:

We used the Grades of Recommendation, Assessment, Development, and Evaluation (GRADE) assessment to evaluate quality of the evidence reported within the included RCTs [67]. Two authors (MP and JEC) independently evaluated the GRADE domains including: risk of bias across studies, inconsistency, indirectness, imprecision, and publication bias to assign a quality of evidence level (i.e., high, moderate, or low) [67].

### Data synthesis:

We conducted meta–analyzes using Review Manager (RevMan) software [68]. For each RCT, we extracted change–from–baseline scores. We selected the the immediate post–intervention assessment as the primary time point for analysis in each study, because these values were the most consistently reported endpoint across trials. We screened selected RCTs to confirm the use of consistent test measures, availability of means and standard deviations, and reported changes in outcomes. We imposed no restrictions on population demographics or the type of dance intervention, allowing for a comprehensive evaluation of the evidence. We included RCTs with similar test measures expressed in consistent units, irrespective of variations in population characteristics or intervention types. We categorized physical symptoms based on sensorimotor constructs measured (e.g., pain, fatigue, gastrointestinal distress). We calculated effect sizes for outcomes using the random–effects method and expressed as standardized mean differences (SMD), 95% confidence intervals (CI), the I^2^ statistic to quantify the degree of heterogeneity between studies, and the *p*-statistic to assess significance of summary effect.

## Results

### Search results and selections

Of 4,792 articles screened, 16 publications met the criteria for inclusion, [38,69–83] representing the effects of dance intervention on physical sequelae of survivorship as measured within 12 distinct RCTs. The Preferred Reporting Items for Systematic Reviews and Meta–Analyzes (PRISMA) study selection flow diagram depicts works reviewed in Figure 2.

### Risk of bias assessment

Risk of bias in the included studies was low, predominantly, across domains assessed and across studies included (Table 1), indicating methodological rigor. Blinding of participants to the arm they were randomized to participate in was not possible due to the nature of the interventions (e.g., dance training vs. routine treatment); we considered lack of blinding of participants to represent a low risk of bias. Two dance styles, or *techniques*, were studied by multiple research groups: Argentine tango [38,81] and Greek traditional dance [76–78,82]. Eight additional dance techniques were evaluated by a single research group: belly dance [69–71], dance movement therapy [74,75], guang chang wu [73], The Lebed Method™ [80], partnered ballroom survey [79], Latin dance survey [83], and improvisation [72], The Lebed Method™ [84], partnered ballroom survey [82], Latin dance survey [83], and improvisation [85]. While these studies were conducted as RCTs and were assessed as having a low risk of bias, replication by independent research teams has yet to be performed for these techniques.

### Quantitative results

Table 2 lists the publications identified for inclusion in this systematic review and meta–analysis [38,69–82]. We grouped papers by RCT, depicting one RCT study per row to indicate that outcomes were collected from the same study cohort across multiple published papers. For instance, Ho et al. 2016 and Ho et al 2018 report [74,75] different outcomes from one study cohort randomized to dance movement therapy or usual care; thus we group these 2 publications within a single row. Using this organizational scheme, we identified that the 16 publications represented the results of 12 RCTs.

### Study characteristics

The 12 RCT studies identified studied the following types of cancer, with the number of studies per type indicated in parentheses: breast (9) [38,69–71,73–76,80–83] and any cancers (3) [72,77–79]. Two of the 3 trials that enrolled any cancers limited inclusion to women [72,79]. Two studies enrolled all stages of cancer [38,72] while 7 studies limited inclusion to stages 0-III [69–71,73–75,77,78,80,81,83] and 3 did not specify inclusion criteria regarding stage [76,79,82]. Four of the RCTs focused on the chronic phase of survivorship [38,69–71,73,76–81,83] while 2 focused on the active phase of survivorship, including while survivors received chemotherapy (1) [73] and radiotherapy (1) [74,75] treatment. Two RCT enrolled individuals in any phase of survivorship across the continuum of care [73,77,78,80]. An additional 2 trials did not specify inclusion criteria regarding phase [72,82]. Trials were performed in the United States (4) [38,79,80,83], Greece (3) [76–78,82], Brazil (2) [69–72], China (2) [73–75], and Germany (1) [81].

### Interventional characteristics

We identified 9 dance forms that were evaluated to treat the physical effects of cancer via RCT study, to date, with the number of independent research groups to have conducted RCTs on the dance form indicated in parentheses: Greek Traditional (2) [76–78,82], Argentine tango (2) [38,81], dance movement therapy (1) [74,75], belly dance (1) [69–71], a survey of partnered ballroom styles (*ballroom survey;* i.e., Foxtrot, Waltz, Cha–Cha, East Coast Swing) (1) [79], a survey of Latin dance forms (Latin dance survey: i.e., Merengue, Bachata, Salsa, Cha–Cha) [83], guang chang wu (1) [73], The Lebed Method™ - Focus on Healing Through Movement and Dance (1) [80], and dance improvisation (1) [72]. Three dance techniques studied were adapted from a traditional dance form in order to address physical mobility issues specific to breast cancer survivors (Adapted Argentine tango [38], Greek Traditional dance [76], guang chang wu [73] while one dance technique was designed specifically for breast cancer survivors from inception (The Lebed Method™) [80]. Four studies were translated to English from their respective languages for analysis [72,77,82,84].

Across techniques, interventions involved social activity. For instance, Argentine tango, ballroom survey, and Greek traditional dance were practiced in groups and included physical contact with another dancer in a line, circle [76] or couples’ partnership formation [38,79,81]. In terms of the setting in which dance was instructed: 3 started intervention training in a hospital setting then transitioned to a community space [72,74,75] including independent home practice [85]; five were performed entirely in community spaces [38,69–71,74,76,79,83]; and 4 studies did not specify setting. Regarding instructor qualifications, 5 interventions were instructed by dance experts [38,69–71,76,79,81,83] with 2 having received specialized training to ensure safety of participants with medical conditions [38,69–71,76]. Two dance interventions were delivered by certified dance movement therapists (DMTs) [74,75,80] with specialized training in dance instruction for cancer survivors. Two dance interventions were delivered by licensed medical professionals (i.e., nurses, physiotherapists) who had been coached by dance experts in the technique they were to deliver [73] or who taught the class in partnership with a dance expert [69–71]. Three RCTs did not specify instructor qualifications.

The FITT characteristics of dance–based interventions varied among the included studies. *Frequency* of training sessions ranged from once per chemotherapy cycle to 5 times per week [73] with the most common frequency being twice per week [38,72,74,75,77,78,80,83]. *Intensity* of activity was reported to be moderate for 5 trials [38,72,76,79,83] and high for 3 trials [69–71,77,78,82] but was unspecified in 4 trials [73–75,80,81]. Across studies reporting intensity at any level, descriptions were qualitative (e.g., moderate, high) and referred to the intended intensity of the activity design, but no trials confirmed intensity experimentally. The lack of experimental confirmation of intensity level stands to contribute to heterogeneity in outcomes and reproduction of results. *Time* in terms of session ranged from approximately 20 minutes (mean(SD) = 18.4(5.7)) [38] to 90 [74,75] and in terms of longitudinal period of participation ranged from 3 weeks [74,75] to 24 weeks [76] with the most common session duration being 60 minutes [69–72,76–78,80,81,83] and most common period being 6 to 12 weeks [38,74,75,77–83] (Table 2).

### GRADE assessment results

Results of the GRADE assessment are reported in Table 3. Strength of recommendation was assessed to be strong for all outcomes assessed via meta–analysis.

### Outcome effects

We detail specific outcomes used per study in Table 4. The most common measure of physical symptoms was patient–reported physical health (6) [72,73,77,79–81]. Other outcomes included: patient–reported fatigue (4) [69,75,81,82], stress (3) [69,75,78], patient–reported pain (4) [69,74,75,78], physical activity level (4) [38,79,82,83], patient–reported sleep (3) [69,75,81], patient–reported gastrointestinal (GI) dysfunction (2) [77,81], walking endurance (2) [76,79], range of motion (2) [71,80], anthropometrics (2) [76,83], respiration (1) [77], sexual function (1) [70], hand grip strength (1) [76], and dual–task function (1) [38].

Of the outcomes reported, meta–analysis was possible for the effects of dance intervention on the following outcomes, with number of RCTs included in the meta–analysis indicated in parenthesis after the outcome: physical function PRO (6), fatigue PRO (4), physical activity level (4), sleep PRO (3), stress PRO (3), gastrointestinal function PRO (2), and 6 minute–walk test (6mwt) (2). Details required to enable meta–analysis were reported within the published paper or provided through correspondence with the lead author [81,86]. Meta–analysis findings (Figure 3) revealed a significant, favorable effect on patient–reported physical health and a non–significant favorable effect on the remaining 5 outcomes considered. Patient–reported physical health showed a standardized mean difference (SMD) = 1.51, 95% confidence interval (CI) = [0.23, 2.79], *p* = 0.03, I^a^ = 93%; GI dysfunction SMD = 0.53, CI = [−0.27, 1.34], *p* = 0.08, I^a^ = 0%; fatigue SMD = 0.35, CI = [−0.18, 0.88], *p* = 0.12, I^a^ = 57%; stress SMD = 1.43, CI = [−1.48, 4.34], *p* = 0.17, I^a^ = 98; 6-minute walk test of endurance SMD = 0.37, CI = [−1.43, 2.17], *p* = 0.23, I^a^ = 0%; physical activity level SMD = 0.82, CI = [−0.99,2.63], *p* = 0.24, I^a^ = 82%; and sleep SMD = 0.44, CI = [−0.89, 1.77], *p* = 0.29, I^a^ = 83%. Effect sizes represented by SMD values were interpreted as small, moderate, or strong according to Kinney, Eakman, and Graham (2020) [87] as follows: we considered effect sizes from 0.08 to 0.15 as small; effects sizes in the range of 0.19 to 0.36 as moderate; and effect sizes from 0.41 to 0.67+ as large, with effect sizes above 0.67 as particularly strong.

## Discussion

This is the first systematic review and meta–analysis to analyze the effects of dance interventions explicitly on the physical sequelae of survivorship, including chemotherapy treatment effects, providing a focused synthesis of the potential to treat physical symptoms of cancer and cancer treatment non–pharmacologically through dance. Twelve randomized control studies met the inclusion criteria. Pooled estimates indicate support for the effect of dance on patient–reported physical health, GI dysfunction, fatigue, stress, walking endurance, physical activity level, and sleep. Further, results of individual RCTs suggested that dance may have beneficial effect on other physical symptoms of survivorship including, but not limited to, pain and dual–task function. Meta–analysis found that patient–reported physical health outcomes reached significance at the level of *p* < 0.05 and patient–reported GI dysfunction outcomes showed a trend toward significance (0.05 < p < 1.0). Effect sizes were large for patient–reported physical health, GI distress, stress, physical activity level, walking endurance, and sleep and moderate for fatigue.

We interpret this evidence to indicate that dance represents a candidate modality for survivorship care and oncology rehabilitation. Future research must provide more specific definition of NDT protocols, in terms of the FITT model, and establish reproducibility of effect for each interventional dance technique defined within the literature. Toward this end, we recommend applying the Rehabilitation Treatment Specification System (RTSS) [88–90] framework to define the dance techniques that are proposed as potential NDT candidates. The RTSS enables innovators to identify the salient ingredients of successful dance–based intervention, the neurophysiologic mechanisms of action through which dance activity might improve physical health among survivors, and key outcome targets for which dance serves as effective physical medicine modality within oncology rehabilitation and survivorship care.

**Figure 1.**
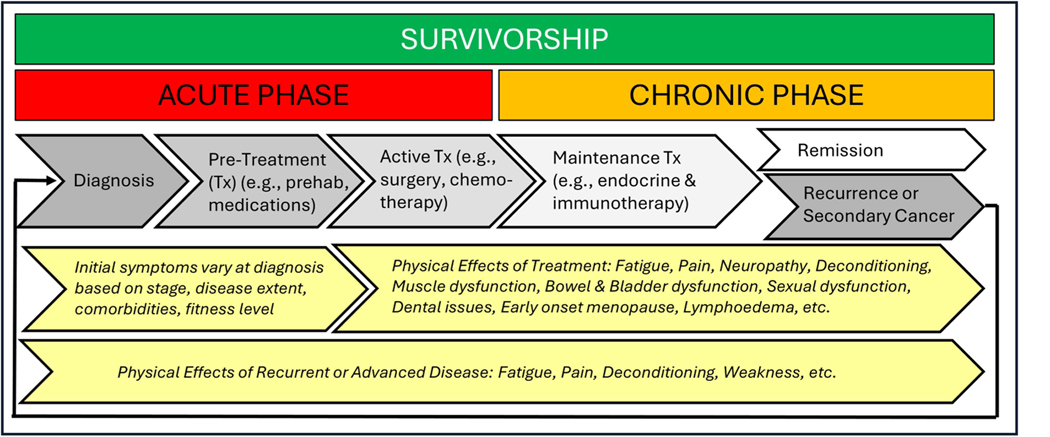
Schematic depicting the scope of medical care for the physical effects of cancer from initial diagnosis throughout the cancer care continuum. Abbreviations: Tx = Treatment; prehab = prehabilitation (i.e., process of preparing patients for medical procedures by improving physical and mental health).

**Figure 2.**
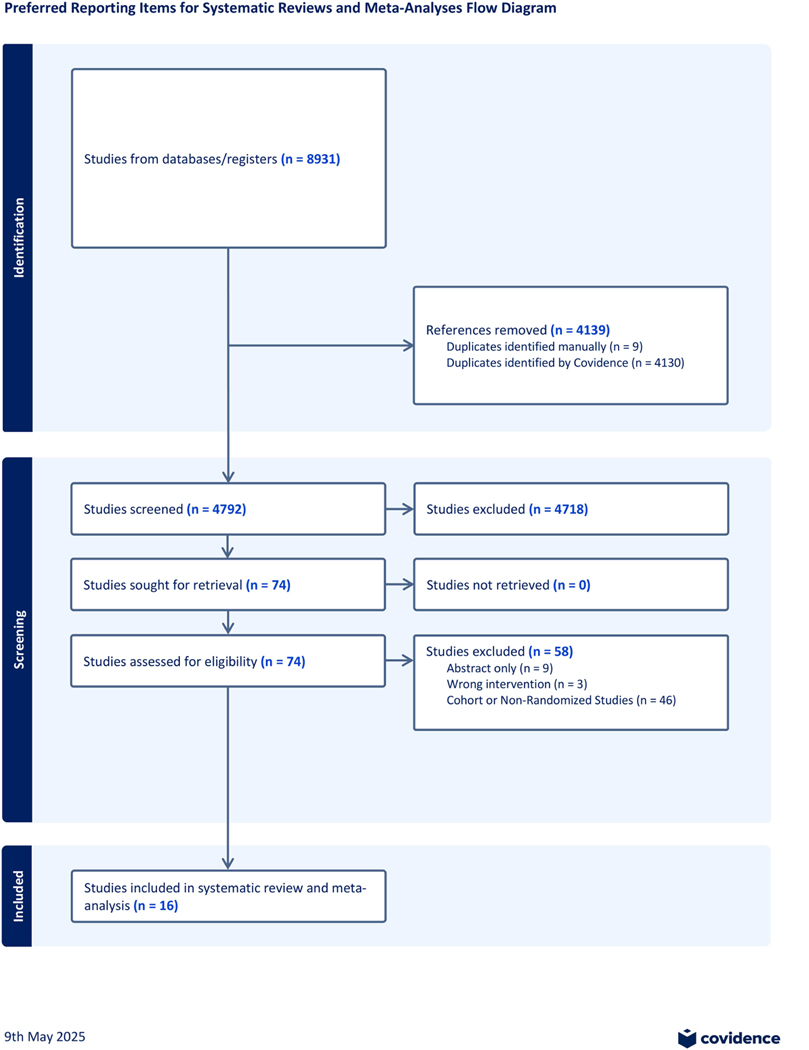
PRISMA Diagram for Neurologic Dance Training (NDT) as Physical Medicine for Survivors of Cancer: A Systematic Review and Meta–Analysis.

**Figure 3.**
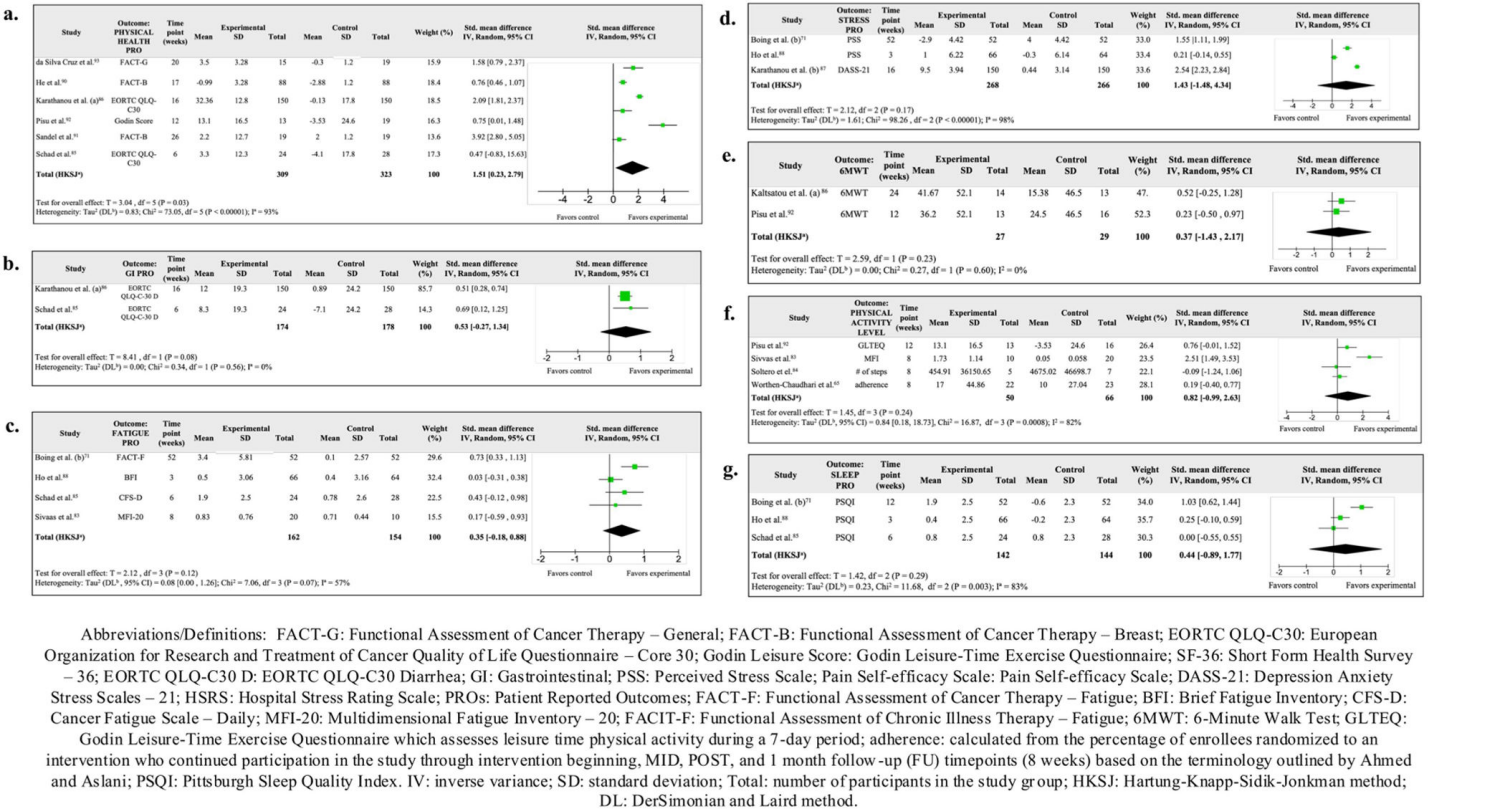
Meta–Analysis of physical outcomes among cancer survivors participating in dance–based intervention: a. patient–reported physical health, b. GI dysfunction, c. fatigue, d. stress, e. walking endurance as measured by the 6MWT, f. physical activity level, and g. sleep.

Most trials evaluated physical outcome targets among survivors with cancer stage I through III (Table 2) who were in the chronic phase of disease (Figure 1, Table 2). Papers included in this review, however, established feasibility and preliminary effect of dance–based intervention delivered during the acute phase of cancer treatment and among survivors with stage IV cancer, including metastatic disease. Additionally, most interventions delivered training in community settings and evidence of efficacy came from a host of countries in North America, South America, Asia, and Europe. We interpret these results to support the premise that dance–based intervention represents a candidate longer–term, community–based pathway to address the physical sequelae of cancer across the continuum of care and among survivors of any stage living around the world.

We identified the common FITT characteristics of dance–based intervention for survivors to be: twice a week frequency, class duration of up to 60 min, and interventional period of at least 6 weeks. All studies included defined type of activity in terms of dance technique, but medically relevant modifications to the technique were less commonly reported. More than a third of studies neglected to report intensity level. Where reported, intensity most often was designed to progress from low to moderate with 3 studies including bouts of high intensity. Despite a lack of confirmation that participants achieved intended intensity levels, we conclude that most interventions studied delivered at least a moderate–intensity level of activity and that dance is a feasible interventional design through which to deliver high–intensity interval training (HIIT) to survivors. Future studies of dance as physical medicine for survivors should verify intensity level by collecting heart rate or a surrogate measure such as rating of perceived exertion from participating survivors [38] and report intensity in addition to frequency, time, and type of dance intervention.

Per the RTSS framework [88–90], we considered what mechanisms of action were indicated by these common FITT characteristics. Based on our finding that outcomes improved within 6 weeks of intervention, we hypothesize that one mechanism of action through which physical symptoms improve with dance practice is *skill acquisition*, the process of gaining new abilities and competencies through experience, practice, and learning [91]. Quantitative studies have shown that six to eight weeks of skill acquisition training induces neuroplasticity as evidenced by changes in central myelin [91,92], functional connectivity [93], and other spinal and supraspinal adaptations [94]. Therefore, future studies should consider the impact of dance–based interventions as skill acquisition paradigms and measure effects on the central nervous system to verify physiologic mechanisms of action. In terms of activity intensity, interventions studied were designed to deliver a moderate level of intensity and to last for up to 60 minutes per session. Because moderate intensity for 60 minutes of activity fits the guidelines for aerobic condition [42,95], we hypothesize that aerobic conditioning is another driver of physical symptom change due to dance–based intervention. Training effects of aerobic conditioning include mechanisms such as cardiovascular adaptations, enhanced oxygen and glucose metabolism, increased amounts of mitochondria and myoglobin within muscle fibers, reduced stress reactivity, and increased blood flow [96]. Of note, the dance forms of Greek traditional dance and belly dance purportedly delivered HIIT, which drives unique neuromuscular adaptations in terms of power production [97]. Finally, the neurophenomenon of *auditory–motor entrainment* is likely stimulated through dance–to–music, thus representing another potential mechanism of action in which a periodic auditory stimulus potentiates neuromotor activity [98]. These hypothetical mechanisms of action provide potential outcomes of interest for future studies of dance as a physical rehabilitation modality.

Another common characteristic of interventions was social engagement. The included literature under-reported the exact nature of social engagement per intervention. For instance, studies underreported the number of participants per group and the ratio of participants to volunteers/staff. Despite this lack of detailed description, we postulate that social engagement is a salient ingredient of dance–based intervention. Potential mechanisms of action linking social engagement to improved health outcomes include motivation to participate [6], improved dyadic trust [79], and reduced stress reactivity [36]. Furthermore, several techniques studied, such as Greek traditional and partnered dance, involved physical connection to other participants, which might deliver some strength and/or perturbation training through the force dynamics created between dancers [99]. Social engagement has proven to be a salient ingredient of exercise–based intervention for survivors [100–102], therefore, future study of social engagement associated with dance–based intervention is warranted.

Instructor qualifications are relevant to reproducing NDT protocols and to the application of dance practices as community–based pathways to survivorship wellness. Ten out of 12 RCTs analyzed specified basic qualifications of instructors, enabling us to make some generalizations about preparatory requirements. Instructors always were prepared with experience in the dance technique they were teaching, and almost always were prepared with safety training in physical activity instruction among individuals with the mobility limitations of the enrolled populations. For instance, most studies among breast cancer survivors were taught by dance experts who had trained with licensed or certified medical exercise experts to learn how to protect the shoulder joint [38,73,76,80], a common area of mobility limitation among breast cancer survivors who have received surgical and radiological cancer treatment affecting the tissues around the shoulder [19]. We interpret this finding as initial evidence that dance–based intervention can be safe and efficacious for survivors when taught by dance experts who have received safety training to address the anticipated mobility limitations of the enrolled population. This further supports our premise that community–based instruction and programming may be a viable pathway to physical wellness for survivors. To increase clarity and strength of evidence regarding qualifications of instructors of dance delivered as physical medicine modality, we recommend that future studies detail the preparatory training of dance instructors working with individuals with movement disorders, as exemplified by existing publications documenting adaptation of partnered Argentine tango as physical activity practice for individuals with Parkinson Disease [103]. Important aspects to report include structured training in falls prevention as well as specific adaptations of traditional dance steps for populations with neural deficits. Ideally, leaders in the emerging field of NDT would develop certification training programs for dance professionals who seek specialized knowledge in safe intervention facilitation among individuals with medical constraints. Such certification programs should cover the basics of safety and effectiveness as do certification programs in the field of neurologic music therapy (NMT) [8,64].

As depicted in Table 2, many studies reviewed compared dance as an alternative to usual care, a logical first step to establish effect of dance as a cancer rehabilitation modality. Once effects of dance versus usual care are more clearly defined, comparative effectiveness studies of dance versus other promising physical activity interventions, such as exercise [6,38], will be needed. One RCT identified in this review compared dance to exercise [38], providing an example of what such comparative effectiveness trials could look like. However, to compare dance to exercise or other activity (e.g.,physical therapy), NDT protocols need to be clearly defined in terms of the FITT principles. Future studies should report these characteristics in the level of detail established by authors of the international consensus statement on exercise for cancer survivors [42]. This published consensus statement defines specific FITT characteristics of exercise to improve physical symptoms of cancer including cancer–related fatigue, physical function, and health–related quality of life as well as specific adverse treatment effects such as chemotherapy–induced neuropathy [42], setting the standard of reporting for other physical activity–based interventions and thereby supporting the development of umbrella reviews of the scientific evidence that have great potential to translate to clinical practice [104]. Finally, clinical trials of rehabilitation modalities should follow the 2017 CONSORT guidance for clinical trials of nonpharmacological interventions including reporting how adherence is assessed, reporting eligibility criteria for centers performing the intervention and mid–trial updates developed during trial implementation among participants living with the condition being studied such as changes to the intervention or adherence–motivation techniquest [105].

We identified gaps in knowledge within the existing literature. For instance, no studies explored the impact of social context by studying class size as an independent variable. The number of individuals trained together at one time within group intervention is a variable of interest within research of community–based intervention. Group class size potentially mediates a range of outcomes including cost–effectiveness, feasibility of outcome measurement in groups, and outcome effects attributable to social interaction experienced within the intervention. Additionally, future studies should report survivor adherence and participation rates. Habitual participation in physical activity mediates long–term relief from cancer effects [42] and represents a known challenge for survivors [106,107]. Therefore, participation rate data are needed, in addition to dose response and effect size data, to support advances in physical activity design that promotes long–term participation and associated symptom relief. We noted a dearth of research into the rehabilitative effects of dance on neurologic complications of cancer treatment such as chemotherapy–an area of demonstrated need in terms of the long–term consequences for both physical and cognitive health [5]. Furthermore, most studies focused on survivors who were women, by limiting enrollment to women or by limiting enrollment to survivors with breast cancer, a form of cancer that affects women more frequently than men. Therefore, evidence for the effect of dance–based intervention on the physical sequelae of cancer is limited largely to survivors who are women. We speculate that this limitation was imposed because the authors considered women more likely to participate in dance interventions than men. However, the literature examining dance–based intervention for Parkinson Disease contradicts this assumption and indicates high attendance rate and responsivity of men to Adapted Argentine Tango [108], Irish set dance [109], the “Dance Well” technique developed in Italy [110], and the Dance for PD® technique developed by dancers from the Mark Morris Dance Group in the US [111]. More research is needed to establish feasibility and effect of dance–based intervention among survivors who are men.

### Limitations:

In addition to identifying gaps in the existing literature, we found that lack of detail regarding intervention and study design, lack of clinical trial progression from Phase I (i.e., feasibility) through Phase II (i.e., efficacy) to Phase III (i.e., effectiveness) per dance intervention studied, heterogeneity across characteristics reported per intervention, and small sample sizes per study within the current literature limited the conclusions we could draw around subgroup analysis or dose effects of dance–based intervention. General elements of dance–based activity dose that were underreported included descriptions of the FITT criteria, instructor qualifications, delivery setting, and related adverse events. Additional aspects of intervention design that were underreported included musical cadence, level of attention to rhythmic movement entrainment, and quality of sound (e.g., live music, surround sound); these variables are relevant to reproducing interventions and to differentiating the effects of dance and/or music versus exercise. To facilitate the translation of dance from recreational activity to care modality, the following additional information is needed. Studies should report the maximum number of participants with movement disorders trained within group intervention settings because this is relevant to the interventions’ scalability and potential broader applicability. Additionally, understanding the type and prevalence of movement disabilities within these participant groups would offer insights into the medical challenges addressed. Equally important is the ratio of participants to research volunteers, as this could influence the intensity, deliverability, and safety of the interventions. Moreover, the inclusion of caregivers in the studies is a significant aspect that warrants attention. Specifically, knowing whether caregivers were actively involved in experiencing the intervention themselves, assisting in the delivery of the intervention, and/or serving as guards to ensure safety during intervention could provide valuable context for interpreting the results. Equally important is the lack of consistency in follow–up reporting, which prevented us from pooling long–term outcomes. Future studies should prioritize standardized follow–up assessments to better capture the sustained impact of dance interventions. Finally, we limit this review to consideration of dance forms performing movement to music. Although dance behavior does not inherently require musical accompaniment, when movement is performed with attention to music, then the practice can certainly be classified as dance such that movement–to–music intervention represent the most concrete example from which to start consideration of dance as physical medicine modality. Nonetheless, forms of dance that are performed in the absence of external auditory stimuli stand to train integration between non–auditory senses, such as proprioceptive, visual, and/or vestibular systems (i.e. graviception [112] and require further study. Future research evaluating dance as a nonpharmacologic treatment option should address these gaps in reporting of dance–based intervention and study design. Toward this end we include recommendations for future research in Supplementary Table B.

## Conclusion

Implications for practice: Evidence supports dance as a safe and effective nonpharmacologic, community–based treatment modality through which to alleviate the physical symptoms of cancer disease progression and treatment among survivors when practice occurs at a frequency of twice a week, intensity level reaches at least moderate, time per session is 20−60 minutes, and time per intervention period is 6−12 weeks. Interventions may be instructed by licensed medical professionals with specialized dance training or by dance instructors who have undergone specialized safety training; more work is needed to define the characteristics of adequate training for either type of instructor. Furthermore, more reproduction of results per technique is required to substantiate subgroup analysis of specific adapted dance techniques. Finally, there is opportunity for further research to define the effects of dance–based intervention in the acute phase of cancer survivorship, among survivors who are men, and in terms of underlying neurophysiologic mechanisms of action.

This review establishes that dance–based intervention can serve as a physical medicine modality for survivors. We name this emerging field of augmentative medical expertise Neurologic Dance Training (NDT). Future studies should continue to evaluate NDT intervention design relative to usual care, reporting interventions characteristics in terms of the FITT model, use of musical accompaniment, characteristics of social engagement, and dance instructor preparation to work with individuals with the physical sequelae of cancer disease progression and treatment.

## Supplementary Material

Supplementary Table A

Supplementary Table B

Supplemental data for this article can be accessed online at https://doi.org/10.1080/28352610.2025.2594717.

## Figures and Tables

**Table 1.** Risk of bias (ROB) assessment of included trials.

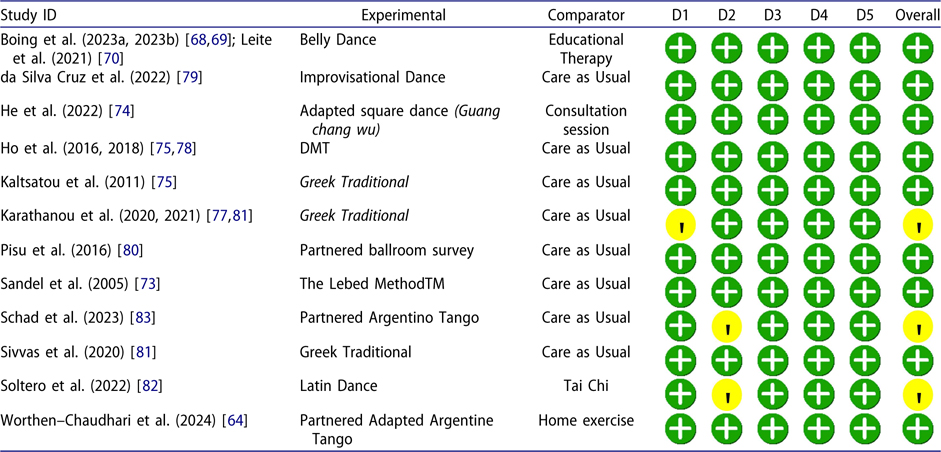

Note: D1 = Randomization process; D2 = Deviations from the intended interventions; D3 = Missing outcome data; D4 = Measurement of the outcome; D5 = Selection of the reported result. = low risk of bias; = some concerns about potential for bias; = high risk of bias.

**Table 2.** Characteristics of randomized controlled studies found to have measured physical health outcomes among cancer survivors undergoing dance intervention.

Study publications	Frequency	Intensity	Time (min)	Type (dance form)	Adaption of existing dance form?	Instructor background	Delivery Setting (communityor hospital)	Country	Comparison type (Usual care or Active)	n	Age mean (SD) years	Related Adverse Events	Cancer Type	Stages of cancer included	Phases of survivorship addressed
Boing et al. (2023a, 2023b) [68, 69]; Leite et al. (2021) [70]	3x/week for 16 weeks	Low to high	60	Belly dance	Not specified	1 physio–therapist and 1 exercise science professional with previous experience in dance	Community cancer support center	Brazil	Usual care with education sessions (Note: third intervention of mat Pilates was not analyzed here)	74	Not reported	None	Breast	Stage 0-III	Chronic
da Silva Cruz et al. (2022) [79]	2x/week for 20 weeks	Low to	moderate	60	Improvisation	Not specified	Not specified	Hospital and community cancer support center	Brazil	Usual care (wait list)	48	53.8 (11.8)	None	Any female	survivors
Stages I–IV	Not specified														
He et al. (2022) [74]	1 session per chemo cycle then 5x/week for 16 weeks	Not specified	30	Guang chang wu (English translation: “public dance in the square”)	Yes: adapted for breast cancer	Nurses trained by dance instructor	Hospital followed by community–based practice in public outdoor spaces	China	Usual care	176	48.15 (9.31)	None	Breast	Stage I–III	Active and chronic (i.e., during and following
chemotherapy) Ho et al. (2016, 2018) [75, 78]	2x/week for 3 weeks	Not specified	90	Dance Movement Therapy	Yes: adapted to emphasize Chinese values of modesty and emotional control	Certified dance movement therapist	Hospital and community cancer support center	China	Usual care	121	50 (7.9)	None	Breast	Stage 0-III	Active (i.e., during radiotherapy)
Kaltsatou et al. (2011) [75]	3x/week for 24 weeks	Low to	moderate	60	Greek	Traditional	Yes: adapted for breast cancer in	Dance instructor	therapist	Community center	Greece	Usual care	27	EXP: 56.6 (4.2)	Not specified
							terms of group member contact	consulting with a physical						CONT: 57.1 (4.1)	
Breast	Stage I–III	Chronic (i.e., 3 months post active treatment completion (mean 2.2 years post))													
2x/week for 8 weeks	Low to high	60	Greek Traditional	Not specified	Not specified	Not specified	Greece	Usual care		300	EXP: 58.1 (8.3) CONT: 59.3 (9.1)	Not specified	Any	Stage I–II	Active and chronic	
Pisu et al. (2017) [80]	1x/week for 12 weeks	Low to	moderate	45	Partnered ballroom: Foxtrot, Waltz, Cha–Cha, East Coast Swing	Not specified	Ballroom dance instructor	Community dance studio	US		Usual care (wait list)	31	57.9 (9.3)	Not specified	Any female	survivors
Any	Chronic (i.e., At least 3 months post completion of primary cancer treatment)															
Sandel et al. (2005) [73]	2x/week for 6 weeks	Not specified	60	The Lebed Method™, Focus on Healing Through	No: designed for breast cancer	Registered dance/movement therapist certified in the Lebed method	Not specified	US	Usual care (wait list)	38	EXP: 59.7 (9.8) CONT: 59.5 (13.3)	None	Breast; limited to those who had undergone surgery	Stage I–III	Active and chronic
				Movement and Dance											
Schad et al. (2023) [83]	1x/week for 6 weeks	Not specified	60	Argentine Tango	Not specified	Tango instructor	Not specified	Germany	Usual care (wait list)	50	60.5 (10.3)	None	Breast	Stage I–III	Chronic
Sivvas et al. (2020) [81]	3x/week for 12 weeks	Low to high	50	Greek Traditional	No: traditional dances selected in collaboration with treating clinicians	Not specified	Not specified	Greece	Usual care (wait list)	30	53.97 (4.04)	Not	specified	Breast	Not specified
Not specified2x/week for 8 weeks	Moderate	60	Latin dance: Salsa, Merengue, Cha–Cha, Bachata	Not specified	Latin dance	instructors	Community cancer support center	US	Tai Chi	13	EXP: 53.2 (9.30)CONT: 49.6 (6.22)	Not	specified	Breast	Stage 0-III
Chronic (2 weeks to 10 years past primary treatment)Worthen–Chaudhari et al. (2024) [64]	2x/week for 8 weeks	Low to	moderate	20	Adapted Argentine Tango	Yes: adapted per Hackney (2010), Worthen–Chaudhari (2024)	Former professional dancer with Certified Medical Exercise Specialist training	Community cancer support center	US	Active (home exercise)	52	61.2 (9.65)	None	Breast	Stage I–IV
Chronic (i.e., > 3 months post chemo)															

**Table 3.** GRADE assessment to evaluate the quality of the evidence reported within the included RCTs.

Intervention	Intervention Outcome	Citations	Risk of Bias (Each Study)1 = no serious limitations2 = serious limitations (−1)3 = very serious limitations (−2)	Risk of Bias (across studies) 1 = no serious limitations2 = serious limitations (−1)3 = very serious limitations (−2)	Inconsistency 1 = no serious limitations 2 = serious limitations (−1) 3 = very serious limitations (−2)	Indirectness 1 = no serious limitations 2 = serious limitations (−1) 3 = very serious limitations (−2)	Imprecision 1 = no serious limitations 2 = serious limitations (−1) 3 = very serious limitations (−2)	Publication Bias 1 = no serious limitations 2 = serious limitations (−1) 3 = very serious limitations (−2)	Quality of Evidence (for each outcome) High Moderate Low Very Low	Strength of Recommendation Strong + Weak + Weak − Strong −	Traffic Light Adoption Green = Do it Yellow = Use with Caution Red = Don’t do it
*Dance*	*Physical Health PRO*	*da Silvia Cruz* [79]	1	1	1	1	1	1	High	Strong +	Green
		*He* [74]	1								
		*Karathanou* [81]	1								
		*Pisu* [80]	1								
		*Sandel* [73]	1								
		*Schad* [83]	2								
	*GI PRO*	*Karanthanou* [81]	1	1	1	1	1	1	High	Strong +	Green
		*Schad* [83]	1								
	*Stress PRO*	*Boing* [69]	1	1	1	1	1	1	High	Strong +	Green
		*Ho* [78]	1								
		*Karanthanou* [77]	1								
	*Fatigue PRO*	*Boing* [69]	1	1	2	1	1	1	High	Strong +	Green
		*Ho* [78]	1								
		*Schad* [83]	1								
		*Sivaas* [81]	1								
	*Sleep PRO*	*Boing* [69]	1	1	2	1	1	1	High	Strong +	Green
		*Ho* [78]	1								
		*Schad* [83]	1								
	*6MWT*	*Kaltsatou* [81]	1	1	2	1	1	1	High	Strong +	Green
		*Pisu* [80]	1								

**Table 4.** Summary of between–group intervention effects of dance–based intervention on physical symptoms.

Reference	Dance technique	Physical Health PRO 6	Fatigue PRO 5	Stress (PRO unless *cortisol) 4	Physical Activity Level 4	Pain PRO 5	Sleep PRO 4	Range of Motion 2
Schad (2023) [83]	Argentine Tango	EORTC–QLQ–-C30: physical function subscale *d* = 0.48 (*p* = 0.09)	CFS–D–total: includes physical, affective & cognitive fatigue *d* = −0.64 (*p* = 0.03*)				PSQI: daytime sleepiness subscale *d* = −0.41 (*p* = 0.16)	
Worthen–Chaudhari (2024) [64]	Argentine Tango				intervention adherence outcome (d = 0.03*)			
Boing (2023a) [68]	Belly dance							
Boing (2023b) [69]	Belly dance		FACTF–total: perception of fatigue (*p* = 0.896)	PSS–total: perceived stress (*p* = 0.429)		VAS: intensity of pain (*p* = 0.400)	PSQI: sleep quality subscale	
(*p* = 0.727) Leite (2021) [70]	Belly dance							Rright shoulder flexion
(*p* = 0.616) Ho (2016) [78]	DMT		BFI: fatigue interference subscale *d =* 0.14 (*p* = 0.43)	PSS–total: perceived stress *d* = 0.33 (*p* = 0.039*)		BPI: pain severity subscale *d* = 0.36 (*p* = 0.035*)	PSQI: sleep disturbance subscale *d* = 0.20	
(*p* = 0.240) Ho (2018) [75]	DMT		BFI–total: fatigue severity and interference with daily function (*p* = 0.43)	PSS–total: perceived stress (*p* = 0.03*)			PSQI: sleep disturbance subscale (*p* = 0.61)	
Kaltsatou (2011) [75]	Greek							
Karathanou (2020) [81]	Greek	EORTC–QLQ–-C30: physical function subscale	EORTC–QLQ–C30: fatigue subscale			EORTC–QLQ–-C30: pain subscale		
Karathanou (2021) [77]	Greek			Dass−21: stress subscale				
Sivvas (2020) [81]	Greek		MFI: general fatigue subscale (*p* < 0.001*)		MFI: reduced activity subscale	(*p* < 0.001*)		
Guang chang wu	FACT–B: physical wellbeing subscale (*p* < 0.001)							
daSilva Cruz (2022) [79]	Improv	FACT–G: physical wellbeing subscale (*p* = 0.117)				VAS: intensity of pain (*p* = 0.003*)		
Soltero (2022) [82]	Latin dance				Physical activity level: # of steps			
Pisu (2017) [80]	Partnered Ballroom survey	SF−36: physical functioning subscale *d* = −0.50 (*p* = 0.55)			GLTEQ–total: physical activity *d* = 0.49 (*p* = 0.21)	SF−36: bodily pain subscale *d* = −0.06 (*p* = 0.87)		
Sandel (2005) [73]	The Lebed MethodTM	SF−36: physical functioning subscale						Involved shoulder summed ROM (abduction, extension, flexion, internal and external rotation)
Schad (2023) [83]	Argentine Tango		EORTC–QLQ–C30: diarrhea subscale *d* = −0.69 (*p* = 0.02*)					
Worthen–Chaudhari (2024) [64]	Argentine Tango				cognitive load: mean high gamma brain activity (*p* = 0.02*) TUGCog: dual–task function mean change (*p* < 0.001*)			
Boing (2023a) [68]	Belly dance					FSFI: pain/discomfort subscale	(*p* = 0.038*)	
Boing (2023b) [69]	Belly dance							
Leite (2021) [70]	Belly dance							
Ho (2016) [78]	DMT							
Ho (2018) [75]	DMT							
Kaltsatou (2011) [75]	Greek	6MWT (*p* = 0.001*)		dynamometer: right hand grip *d* = 1.41 (*p* = 0.001*)				Arm volume: left upper arm circumference *d* = 0.50 (*p* = 0.028*)
Karathanou (2020) [81]	Greek		EORTC–QLQ–C30: nausea–vomiting subscale				EORTC–QLQ–-C30: shortness of breath subscale	
Karathanou (2021) [77]	Greek							
Sivvas (2020) [81]	Greek							
He (2022) [74]	Guang chang wu							
daSilva Cruz (2022) [79]	Improv							
Soltero (2022) [82]	Latin dance							BMI and body fat %
Pisu (2017) [80]	Partnered Ballroom survey	6MWT *d* = 0.34 (*p* = −0.38)						
Sandel (2005) [73]	The Lebed MethodTM							

Abbreviations/Definitions: *d*: Cohen’s D when available; p: p-value when available,

* indicates significance <0.05; EORTC QLQ C30: European Organization for Research and Treatment of Cancer Questionnaire C30; CFS-D: Cancer fatigue scale, German version; PSQI: Pittsburgh Sleep Quality Index; intervention adherence: calculated from the percentage of enrollees randomized to an intervention who continued participation in the study through intervention beginning, MID, POST, and 1 month followup (FU) timepoints (8 weeks) based on the terminology outlined by Ahmed and Aslani; cognitive load variable: measured from high gamma-band activity; TUGCog: Timed-Up-and-Go with cognitive task; FSFI: Female Sexual Function Index, validated in Brazil; FACTF: Functional Assessment of Cancer Therapy – Fatigue instrument, validated in Brazil; PSS: Perceived Stress Scale, validated in Brazil; VAS: Visual Analogue Scale; BFI: Brief Fatigue Inventory assessment scale of fatigue severity and its interference with daily functioning; PSS: Perceived Stress Scale measures respondents’ subjective global experience of stress; BPI: Brief Pain Inventory is a measurement scale of pain severity and its interference with daily functioning; WQ: a written open-ended questionnaire developed by the research team was used; Baseline handheld dynamometer: participants were seated with the forearm in neutral position and the elbow at 90° and the mean of 3 measurements was used for further analysis; Dass-21: Lovibond and Lovibond Depression–Anxiety–Stress Scale 21; MFI: Multidimensional Fatigue Inventory; FACT-B : Functional Assessment of Cancer Therapy-Breast; FACT-G: assesses leisure time physical activity Functional Assessment of Cancer Therapy General; VAS: Visual Analogue Scale; SF-36: 36-Item Short Form Health Survey is a scale of functional health and well-being; GLTEQ: Godin Leisure-Time Exercise Questionnaire which assesses leisure time physical activity during a 7-day period; BMI: body-mass index.
