# Supplementary material for: Neurologic dance training (NDT) as supportive care for the physical symptoms of cancer: a systematic review and meta-analysis
Source: Cancer Surviv Res Care. Author manuscript; Available in PMC 2026 Aug 14. (PMC13470630; doi:10.1080/28352610.2025.2594717)
Supplement: Supplementary file 1 [file NIHMS2189388-supplement-Supplementary_Table_A.docx]

**Supplementary Table B: Search strategy performed in PubMed, Embase and Scopus**

| **Database** | **Full Boolean Search String** | **Number of results as of January 2025** |
| --- | --- | --- |
| PubMed | (dancing[mesh] OR dance therapy[mesh] OR danc*[tw] OR music[mesh] OR music therapy[mesh] OR music*[tw])  AND  (neoplasms[mesh] OR neoplasm*[tw] OR cancer*[tw] OR malignan*[tw] OR tumor*[tw] OR tumour*[tw] OR oncolog*[tw]) | 1,828 |
| Embase | (dancing/exp OR ‘dance therapy’/exp OR danc*:ti,ab,kw,de OR music/exp OR ‘music therapy’/exp OR music*:ti,ab,kw,de)  AND  (‘malignant neoplasms’/exp OR neoplasm*:ti,ab,kw,de OR cancer*:ti,ab,kw,de OR malignan*:ti,ab,kw,de OR tumor*:ti,ab,kw,de OR tumour*:ti,ab,kw,de OR oncolog*:ti,ab,kw,de) | 3,596 |
| Scopus | TITLE-ABS-KEY ( "dancing" )  OR  TITLE-ABS-KEY ( "dance therapy" )  OR  TITLE-ABS-KEY ( "danc*" )  OR  TITLE-ABS-KEY ( "music" )  OR  TITLE-ABS-KEY ( "music therapy" )  OR  TITLE-ABS-KEY ( "music*" )  AND  TITLE-ABS-KEY ( "neoplasms" )  OR  TITLE-ABS-KEY ( "neoplasm" )  OR  TITLE-ABS-KEY ( "cancer*" )  OR  TITLE-ABS-KEY ( "malignan*" )  OR  TITLE-ABS-KEY ( "tumor*" )  OR  TITLE-ABS-KEY ( "tumour*" )  OR  TITLE-ABS-KEY ( "oncolog*" ) | 3,507 |
