# Supplementary material for: Neurologic dance training (NDT) as supportive care for the physical symptoms of cancer: a systematic review and meta-analysis
Source: Cancer Surviv Res Care. Author manuscript; Available in PMC 2026 Aug 14. (PMC13470630; doi:10.1080/28352610.2025.2594717)
Supplement: Supplementary file 2 [file NIHMS2189388-supplement-Supplementary_Table_B.docx]

**Supplementary Table A: Summary of recommendations for future research of the effect of dance as a nonpharmacologic treatment for the physical symptoms of progression and treatment of cancer.**

| **Category** | **Summary Item** |
| --- | --- |
| Theoretical framework | Apply the Rehabilitation Treatment Specification System (RTSS) ^65–67^ framework or other relevant theoretical scaffold to define ingredients, mechanisms of action, and key outcome targets per interventional dance technique. |
| Theoretical framework | Evaluate *skill acquisition,* *aerobic conditioning, auditory-motor entrainment or other aspects of musical engagement,* and *social engagement* as potential mechanisms of action underlying training effects. |
| Theoretical framework | Evaluate dance as a longer-term, community-accessible avenue to wellness. |
| Clinical trial study design | Report adverse events. |
| Clinical trial study design | Once effects sizes relative to usual care are established, conduct comparative effectiveness trials of dance vs alternative non-pharmacologic alternatives (e.g., exercise, physical therapy, acupuncture, neurologic music therapy, etc.). |
| Clinical trial study design | Future trials should report standardized follow-up outcomes to better assess the long-term impact of interventions. To capture physical effects of cancer, these should include clinically-relevant measures (e.g. Total Neuropathy Score revised, Timed-Up-and-Go, grip strength, etc.) in addition to patient-reported outcomes. This would strengthen the evidence base and improve understanding of the sustainability of effects. |
| Clinical trial study design | The limited number of studies with participants in each subgroup (e.g., genre of dance) restricts the ability to perform subgroup analyses. Future research should prioritize this to better evaluate the relative effects of specific dance-based interventions. |
| Intervention design | Define neurological dance training (NDT) protocols in terms of the FITT characteristics (frequency, intensity, time, and type) including confirming intensity level using methods such as heart rate, pulse rate, or rating of perceived exertion (RPE) and distinguishing dose of movement-to-music vs. rest periods of classes. |
| Intervention design | Detail any adaptations made to existing dance forms for purpose of participant safety or intervention effect. |
| Intervention design | Describe delivery setting in which intervention is held (e.g. community based vs. hospital space, inside vs. outside). |
| Intervention design | Detail number of participants per group and the ratio of participants to volunteers/staff. |
| Intervention design | Detail instructor qualifications, including any dance expertise and safety training (e.g., fall prevention training) provided to or required from instructors of dance as medically-relevant physical activity. |
| Intervention design | Detail characteristics of any musical accompaniment including cadence, groove, and syncopation. |
| Intervention design | Detail exertion level reached by participants and verify using tools such as the Borg Rating of Perceived Exertion (RPE). |
| Participants | Include survivors of cancer stage IV including metastatic disease. |
| Participants | Include men. |
| Participants | Report nature of support giver involvement (e.g., experienced the intervention themselves, assisting in delivery of the intervention, served as guards to ensure safety). Consider measuring the effect of intervention involvement on support giver as well as survivor. |
| Participants | Report maximum number of individuals with movement disabilities, as well as type and prevalence of movement disabilities, within participant groups. |
